# *PbMC1a/1b* regulates lignification during stone cell development in pear (*Pyrus bretschneideri*) fruit

**DOI:** 10.1038/s41438-020-0280-x

**Published:** 2020-05-01

**Authors:** Xin Gong, Zhihua Xie, Kaijie Qi, Liangyi Zhao, Yazhou Yuan, Jiahui Xu, Weikang Rui, Katsuhiro Shiratake, Jianping Bao, Shahrokh Khanizadeh, Shaoling Zhang, Shutian Tao

**Affiliations:** 1 College of Horticulture, Nanjing Agricultural University, Nanjing, China; 2 Laboratory of Horticultural Science, Nagoya University, Nagoya, Japan; 3 College of Plant Science, Tarim University, Ala’er City, China; 4 ELM Consulting Inc., St-Lazare, Canada; 5 Eastern Cereal and Oilseed Research Centre, Agriculture and Agri-Food Canada, Ottawa, Canada; 6 State Key Laboratory of Crop Genetics and Germplasm Enhancement, Nanjing Agricultural University, Nanjing, China

**Keywords:** Plant cell death, Arabidopsis thaliana, Cell wall

## Abstract

Programmed cell death (PCD) and secondary cell wall (SCW) thickening in pear fruit are accompanied by the deposition of cellulose and lignin to form stone cells. Metacaspase is an important protease for development, tissue renewal and PCD. The understanding of the molecular mechanism whereby pear (*Pyrus*) metacaspase promotes PCD and cell wall lignification is still limited. In this study, the *Metacaspases* gene family (*PbMCs*) from *P. bretschneideri* was identified. *PbMC1a/1b* was associated with lignin deposition and stone cell formation by physiological data, semiquantitative real-time polymerase chain reaction (RT-PCR) and quantitative RT-PCR (qRT-PCR). Relative to wild-type (WT) *Arabidopsis*, the overexpression of *PbMC1a/1b* increased lignin deposition and delayed growth, thickened the cell walls of vessels, xylary fibers and interfascicular fibers, and increased the expression of lignin biosynthetic genes. Yeast two-hybrid (Y2H), bimolecular fluorescence complementation (BiFC) and GST pull-down assays indicated that the PbMC1a/1b protein physically interacted with PbRD21. Simultaneously, the transient expression of *PbMC1a/1b* and *PbRD21* led to significant changes in the expression of genes and lignin contents in pear fruits and flesh calli. These results indicate that *PbMC1a/1b* plays an important role in cell wall lignification, possibly by interacting with *PbRD21* to increase the mRNA levels of some lignin synthesis-associated genes and promote the formation of stone cells in pear fruit.

## Introduction

Pear (*Pyrus*) is an important fruit commodity around the world, and its quality has great significance for its marketing. Due to their poor quality, the export price of Chinese pears is lower than the market average. Many factors affect the quality of pears, among which stone cells are very important^1^. Sterling^2^ and Ranadive and Haard^3^ described the chemical properties of stone cells and the deposition of lignin in the cell wall. Mature stone cells contain ~18% lignin in pear fruits^4^. Therefore, it is of great economic significance to study the formation mechanism of lignin and reduce the contents of stone cells to improve the internal quality of pear.

Lignin deposited as lamellar additions to cell walls is the essence of stone cells in pear fruits^5^. Programmed cell death (PCD) is very important for organism development and tissue renewal^6–8^. The differentiation of tracheary elements (TEs) and vessel elements involves typical types of PCD, accompanied by the extensive lignification of cell walls^9–11^. Moreover, the secondary thickening of cell walls and PCD can be repressed with the same inhibitor, which indicates that the two processes are closely related^12^.

The morphological and biochemical characteristics of the initiation of cell death include cytoplasmic contraction, nuclear morphological changes, cytochrome C release from mitochondria, DNA fragmentation and the involvement of proteases^13,14^. In animals, the activation of PCD is primarily associated with Cys-dependent Asp-specific peptidase (caspase), and caspase-like enzymes have been found during cell death in plants^15^. Metacaspases (MCs) are caspase-dependent proteases presented in plants, protozoa, and fungi and show low sequence similarity to caspase^16^. MCs and caspase cleave substrates at the C-terminus with different amino acid compositions. MCs cut at arginine (Arg) and lysine (Lys), whereas caspase cuts at aspartic acid (Asp)^17^. MCs are divided into the following two types: type I MCs have an N-terminal prodomain and a zinc finger motif, whereas type II MCs have no prodomain in the N-terminal region but exhibit an inactive short functional pre-region^18–20^.

In *Arabidopsis thaliana*, there are three type I *MCs* (*AtMC1–3*) and six type II *MCs* (*AtMC4–9*)^21^. *AtMC1* positively regulates PCD and has the required caspase catalytic residue, whereas *AtMC2* negatively regulates PCD^21^. *AtMC8* is a positive regulator of biotic and abiotic stresses in PCD^22^. *AtMC9* is expressed in differentiated xylem vessels and is involved in PCD in xylem tubular molecules^23,24^. In pepper (*Capsicum annuum L*.), *Camc9* plays a regulatory role in the process of PCD induced by plant pathogens^25^. Yeast *MC Yca1* regulates PCD induced by oxidative stress, osmotic stress, and various other stimuli^26^. *PttMC13* and *PttMC14* are homologs of *AtMC9* in hybrid aspen (*Populus*). Proteomic studies revealed targets of MCs such as cysteine protease RD21, Tudor staphylococcal nuclease, putative aspartic protease3 (PASPA3), heat shock proteins, and 14-3-3 proteins^27^.

Not all *MCs* family members in pear (*PbMCs*) are related to cell wall lignification in fruit. Therefore, it is necessary to identify *PbMCs* that are associated with cell lignification. The genomic sequencing of *P. bretschneideri* cv. Dangshan Su pear has been completed^28^; however, there are few reports of *PbMCs* functions. The objectives of this study were to identify *PbMCs* family members in pear, determine the gene(s) involved in the lignification of pear fruit cells, use transgenic technology to verify gene functions, and identify the target protein(s) by using Y2H technology. The results will validate the function of candidate genes and provide a theoretical basis for further understanding the relationship among PCD, lignin synthesis, and cell wall lignification.

## Results

### Identification and phylogenetic analysis of *PbMCs* family members

In this study, 15 *PbMCs* genes were found in the Chinese white pear genome database (Table S1). After considering the corresponding E-values, we selected 11 homologous *PbMCs* genes to build a phylogenetic tree showing the relationships of pear and *Arabidopsis* proteins (Fig. S1). The topology of the phylogenetic tree classified the PbMCs proteins into two subgroups, and most clades showed high statistical support (pp > 0.90; bootstrap > 80%). The location on the chromosome, molecular weight and isoelectric point (PI) of the *PbMCs* were provided in Table S2. Eight genes were mapped onto chromosomes 1, 5, 7, 9, 10, and 12, and other *PbMCs* were located on scaffold contigs.

### Contents of stone cells and lignin in pear fruits during fruit development

**Fig. 1:**
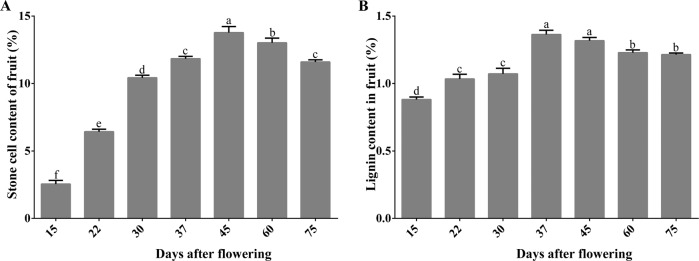
Stone cell content (**a**) and lignin content (**b**) of the fruits during the development of ‘Dangshan Su’ pear. Different lowercase letters on a column indicate that the treatment differs significantly at the 1% level

We determined the contents of stone cells and lignin in pear fruits from 15 to 75 days after flowering (DAF) (Fig. 1) because extensive lignin deposition occurred with stone cells in the early developmental stages^29^. From 15 to 45 DAF, the contents of stone cells increased rapidly, reaching the highest level (13.8%) at 45 DAF, and gradually decreased thereafter (Fig. 1a). The lignin content reached a peak (1.4%) at 37 DAF before the peak of stone cells (Fig. 1b).

### The expression pattern of *PbMC1a/1b* was consistent with the changes in stone cells and lignin contents

**Fig. 2:**
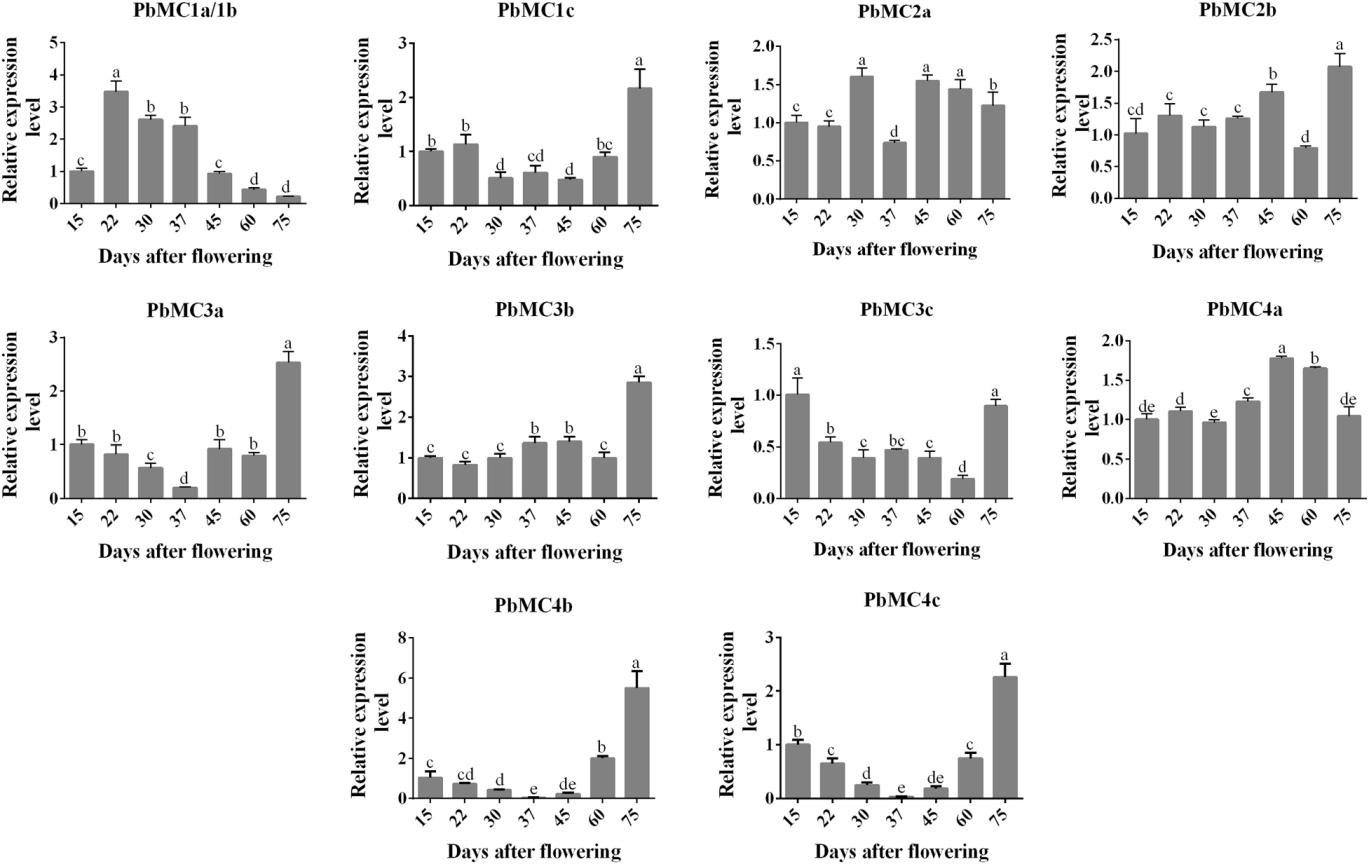
Relative expression patterns of *PbMCs* during the development of ‘Dangshan Su’ pear. Different lowercase letters on a column indicate that the treatment differs significantly at the 1% level

The expressions of *PbMCs* were measured in different pear tissues (Fig. S2). *PbMC1a/1b*, *PbMC2b*, *PbMC3c*, *PbMC4b*, and *PbMC4c* were expressed constitutively in all tissues, whereas the expressions of other *PbMCs* were tissue specific. *PbMC3a* was highly expressed in the roots and pollen, and *PbMC4c* was highly expressed strongly in the roots, leaves, and pollen. *PbMC1c*, *PbMC2a*, *PbMC3b,* and *PbMC4a* were not significantly expressed in any tissues of pear. The expression profiles of *PbMCs* during fruit development were also analyzed (Fig. 2). *PbMC1a/1b* maintained a high expression level from 22 to 37 DAF, was consistent with the trend of stone cell and lignin contents, whereas the other *PbMCs* exhibited inconsistent expression patterns.

### Overexpression of *PbMC1a/1b* increased lignin deposition in transgenic *Arabidopsis* plants

*PbMC1a/1b* may induce PCD by playing a key role in cell wall lignification, and we, therefore, generated transgenic *Arabidopsis* plants that overexpressed *PbMC1a/1b*. In the T1 generation, six hygromycin-resistant transgenic plants were identified (Fig. S4a). Lines OE9, OE10, and OE15 exhibited higher levels of *PbMC1a/1b* expression and were selected for further analyses (Fig. S4b). DNA was extracted again in the T2 generation for identification (Fig. S4c).

**Fig. 3:**
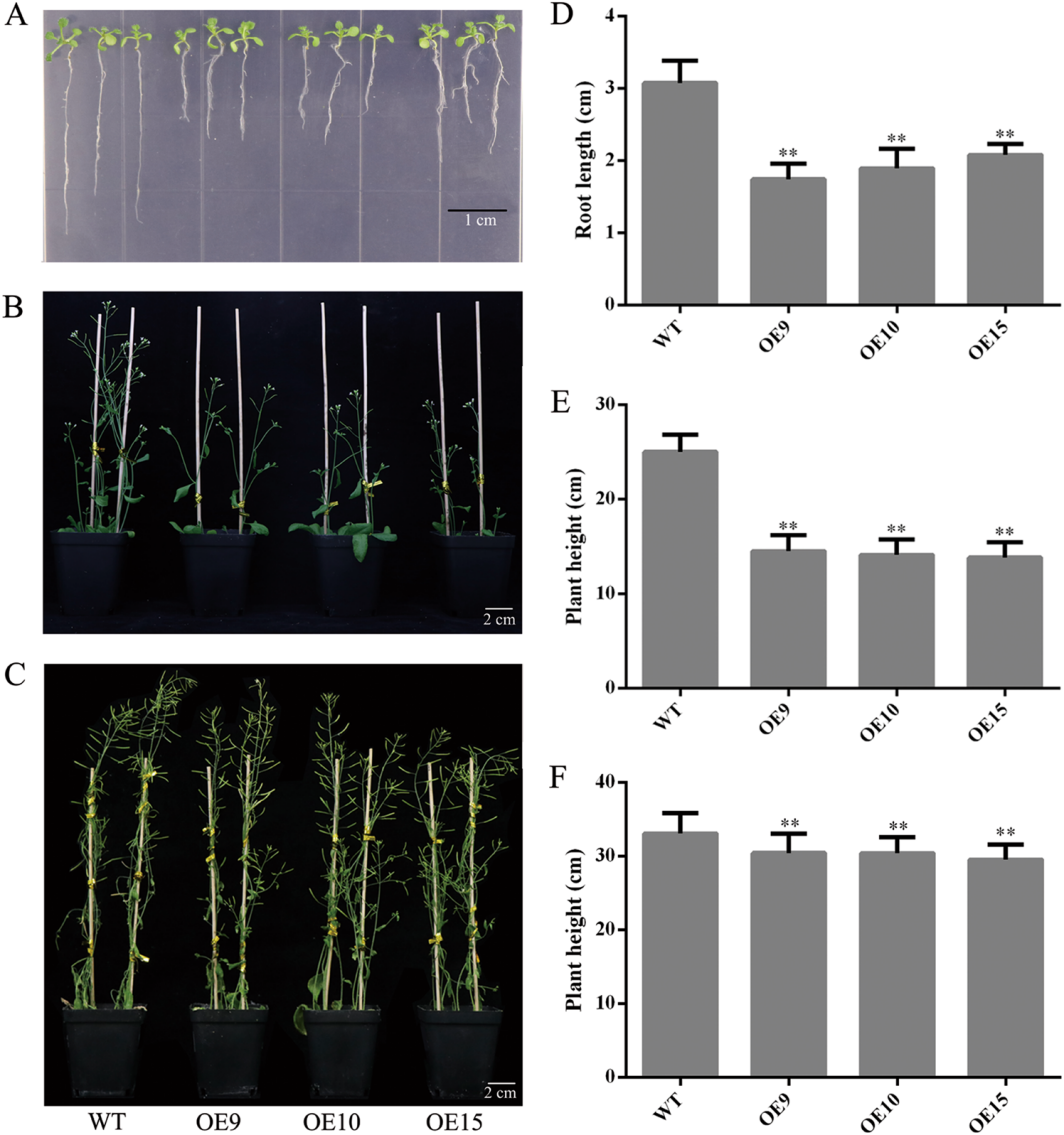
**a**–**c** WT and transgenic plants grown for 7 d (**a**), 5 weeks (**b**) and 8 weeks (**c**) under a long-day photoperiod. Root lengths at 7 days (**d**), plant heights at 6 weeks (**e**) and 8 weeks (**f**) (**p < 0.01)

When the T3 generation was homozygous, OE9, OE10, and OE15 were used for plant phenotype examination. Compared with the WT (3.1 cm), the root lengths of transgenic plants were significantly shorter at the seedling stage (1 week old) and were reduced by an average of 1.17 cm (**P < 0.01) (Fig. 3a, d). During the flowering period (5 weeks old), the growth rates of the transgenic plants were slower, and the plant height of OE15 was the shortest compared to the WT (**P < 0.01) (Fig. 3b, e). At the mature stage (8 weeks old), the transgenic plants were an average of 2.94 cm shorter than the WT (Fig. 3c, f). Furthermore, the stems of the WT inflorescences grew faster, and the overexpression of *PbMC1a/1b* delayed the growth of transgenic *Arabidopsis* plants.

**Fig. 4:**
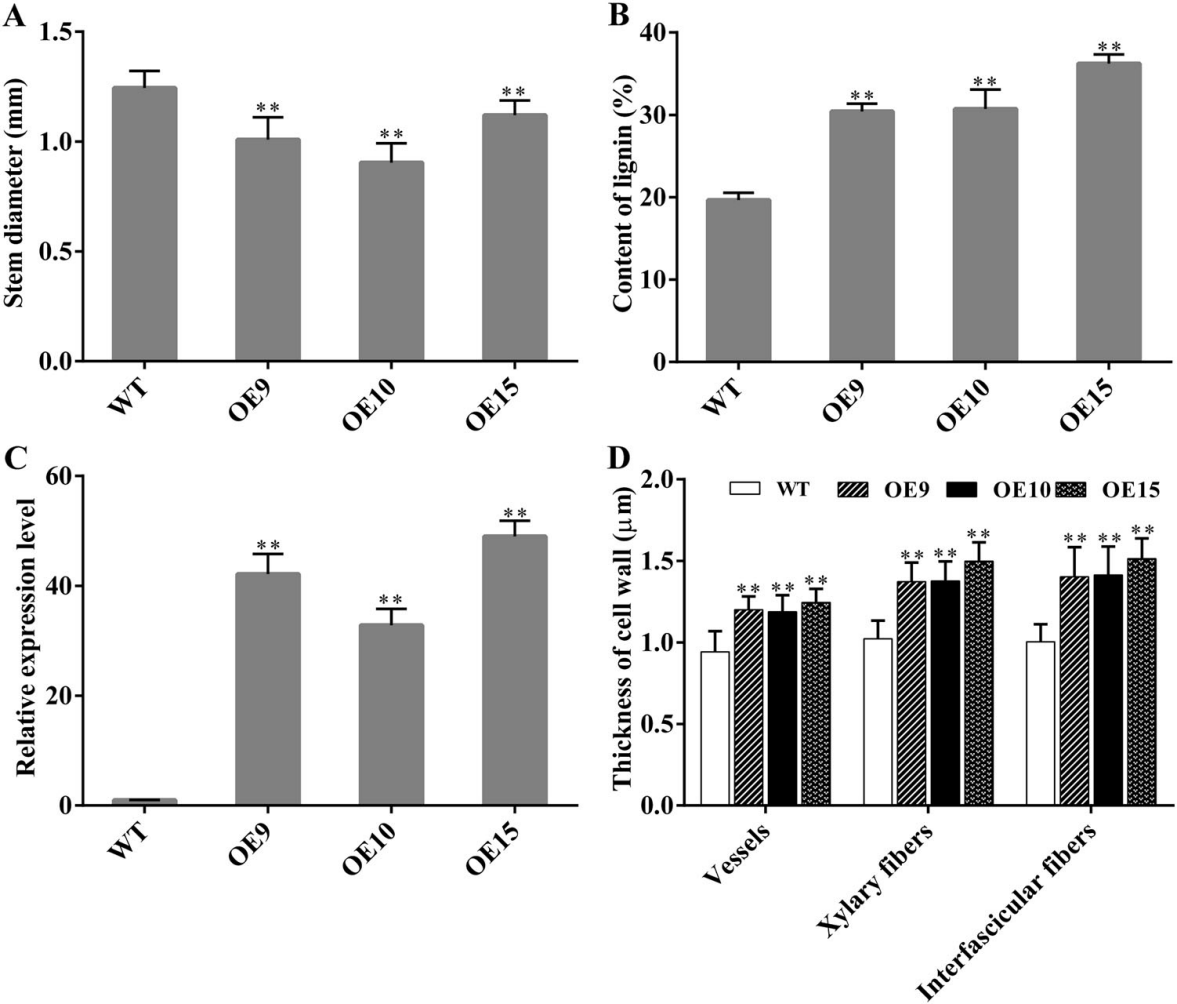
Stem diameters (**a**), lignin contents of stems (**b**), relative expression patterns of *PbMC1a/1b* in stems (**c**), cell wall thicknesses of vessels, xylary fibers and interfascicular fibers (**d**) in WT and transgenic plants (**p < 0.01)

**Fig. 5:**
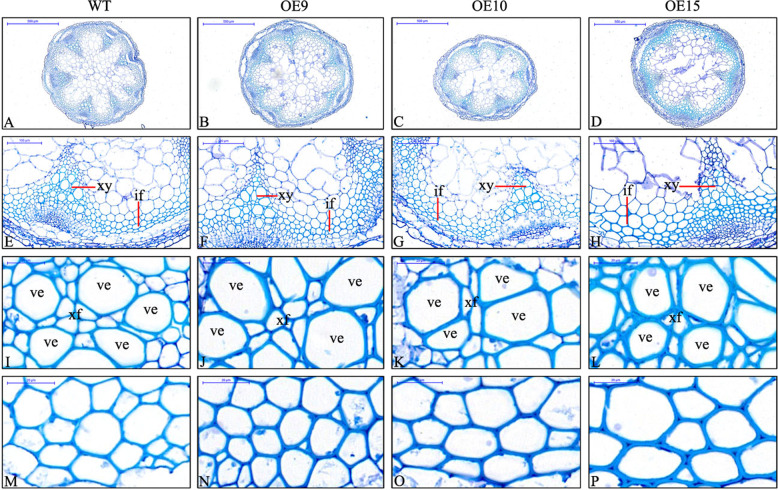
Sections stained with toluidine blue, showing the variation of cell walls in *Arabidopsis* with *PbMC1a/1b* overexpression. **a**–**d**: stem cross-sections, **e**–**l**: xylem, (M-P): interfascicular fiber. if: interfascicular fiber; xy: xylem; ve: vessel; xf: xylary fiber

Interfascicular fibers and xylem cells are the main stem tissues supporting the erect growth of inflorescences^30^, and sections of stems were stained with toluidine blue to observe the changes in cells. The stem diameters of the transgenic plants were significantly smaller than those of the WT (Figs. 4a and 5a–d). The lignin contents of the stems of the transgenic plants were higher than those of the WT, and OE15 was the highest (36.2%), which was 16.57% higher than those of the WT (Fig. 4b). Concurrently, the relative expression of *PbMC1a/1b* was more than 30 times greater in the transgenic stems than in those of the WT (Fig. 4c). There were no changes in the morphology of vessel cells in the transgenic lines (Fig. 5e–h), but the cell wall thicknesses were increased significantly in vessel elements (28.7%), xylary fibers (44%) and interfascicular fibers (36%) compared to the WT (Figs. 4d and 5i–p). These results indicated that *PbMC1a/1b* promoted lignin deposition and cell wall lignification.

**Fig. 6:**
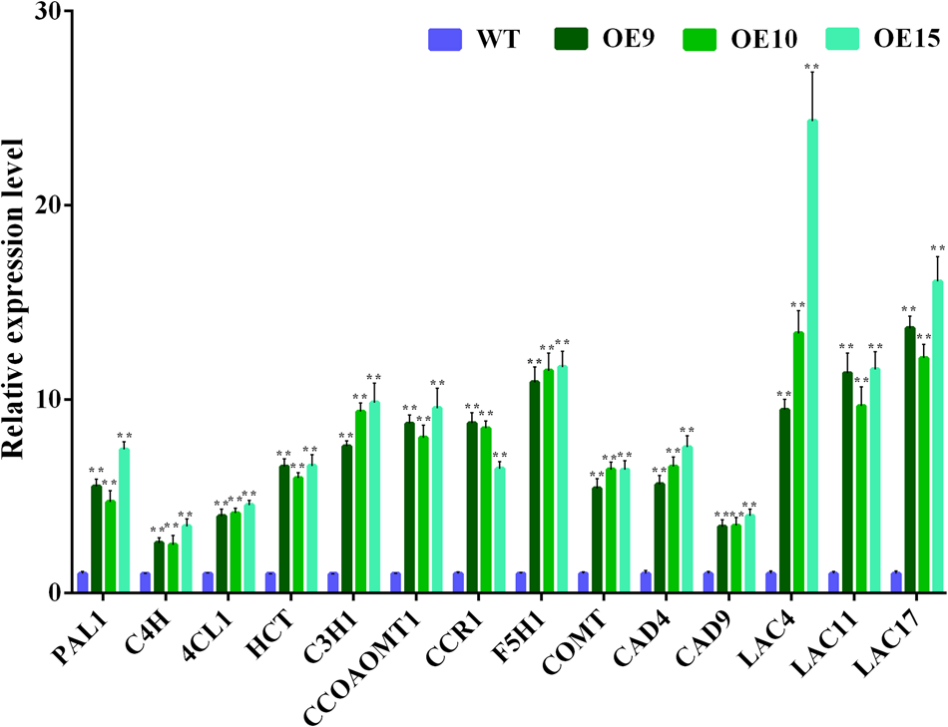
Relative expression patterns of lignin biosynthetic genes in *PbMC1a/1b* transgenic *Arabidopsis* plants (**p < 0.01)

The expression levels of genes are related to the synthesis of lignin in SCWs^31–33^. To elucidate the molecular mechanism of *PbMC1a/1b* in lignin synthesis, the expression patterns of 14 lignin biosynthetic genes, including *4CL*, *C3H*, *C4H*, *CAD*, *CCoAOMT*, *CCR*, *COMT*, *F5H*, *HCT*, *LAC,* and *PAL*, were also analyzed in stems (Fig. 6). The transcription levels of these genes in transgenic plants were significantly higher than those in WT plants, and *LAC4*, *LAC11*, and *LAC17* exhibited the highest expression levels. These genes may synergistically promote the synthesis of lignin in the stems, in which *LAC4*, *LAC11*, and *LAC17* may play key roles.

### PbMC1a/1b physically interacted with PbRD21

**Fig. 7:**
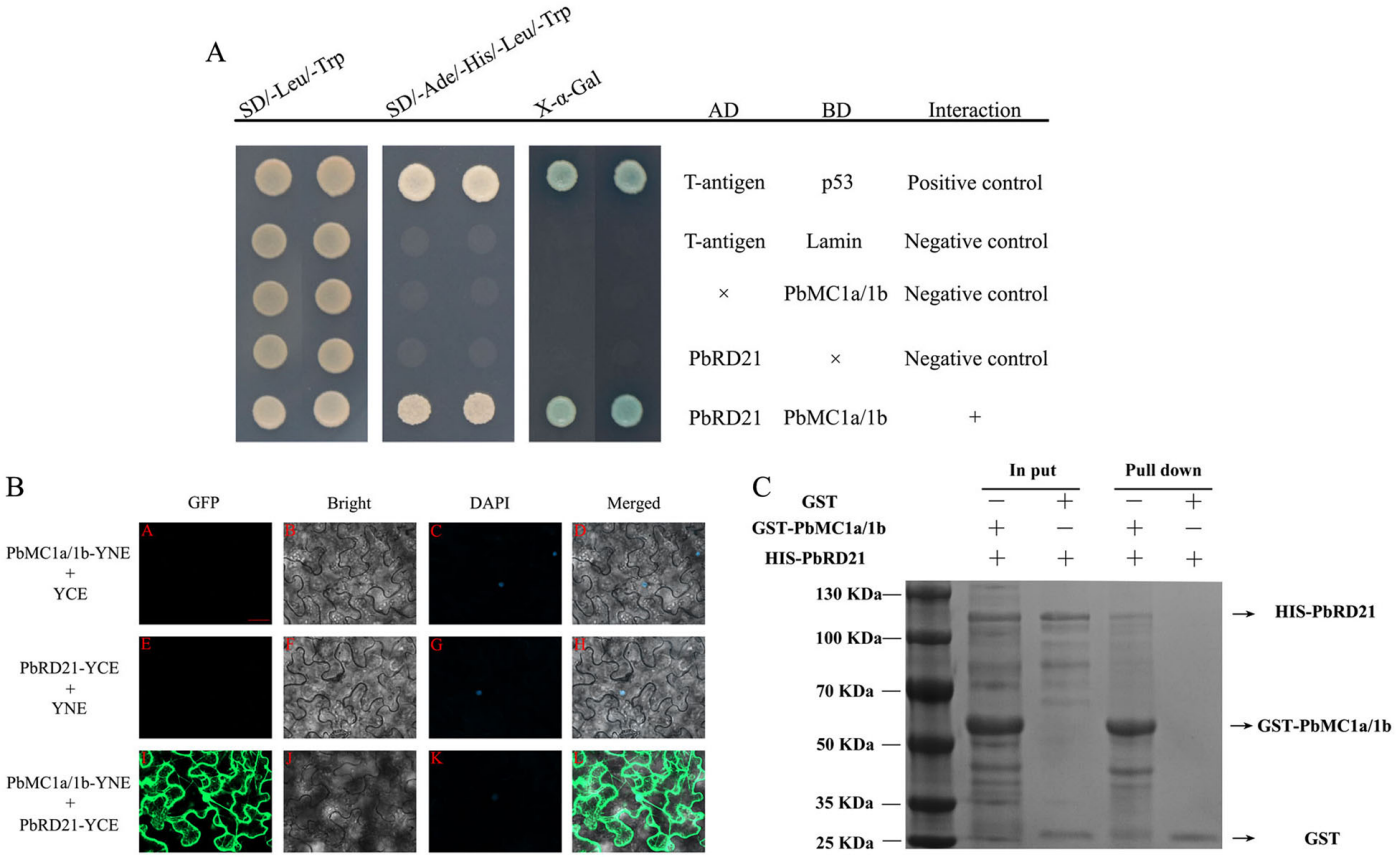
**a** Interaction between PbMC1a/1b and PbRD21 verified by Y2H analysis. Selective growth of the Y2H system was performed by using the X-gal activity assay. Yeast cells were cotransformed with pGAD-PbRD21 + pGBKT7 and pGBK-PbMC1a/1b + pGADT7. pGADT7-T + pGBKT7-Lam was used as a negative control, and pGADT7-T + pGBKT7-53 was used as a positive control. **b** BiFC assay using *Nicotiana benthamiana*. The negative controls were *PbMC1a/1b*-YFP^N^ + YFP^C^ and *PbRD21*-YFP^N^ + YFP^C^. All images were taken by confocal microscopy. Bar: 40 μm. **c** Interaction assay via GST pull-down analysis. PbMC1a/1b and PbRD21 fused with GST and HIS tags were mixed and passed through a glutathione column (binding GST tag). After elution with pull-down binding buffer, the samples were separated by SDS-PAGE. GST alone was used as the negative control

Cysteine protease is a putative target of MCs, and the three peptides of RD21 are highly expressed in the phloem, vascular cambium, and xylem tissues^27^. Y2H was performed to identify whether PbRD21 may function in a complex with PbMC1a/1b. Only the positive control yeast cells and co-transformants (pGBK-PbMC1a/1b and pGAD-PbRD21) grew normally on SD/-Ade/-Leu/-Trp/-Ura medium, whereas the negative controls did not survive (Fig. 7a). Furthermore, the positive control and co-transformants exhibited blue coloration after X-α-Gal staining.

To verify the possibility of their interaction, the subcellular localizations of PbMC1a/1b and PbRD21 were identified. Control GFP was distributed evenly throughout tobacco cells, while the PbMC1a/1b-GFP fusion protein was located in the cytoplasm and nucleus, and PbRD21-GFP was only found in the nucleus (Fig. S3). Through subcellular localization analysis, both PbMC1a/1b and PbRD21 were found in the nucleus, which may indicate the possibility of their interaction.

The results of Y2H analysis were verified by BiFC and GST pull-down assays. Green fluorescence was observed in tobacco cells harboring the PbMC1a/1b-pSPYNE and PbRD21-pSPYCE vectors. However, no green fluorescence was observed in cells transformed with the negative control vectors (PbMC1a/1b-pSPYNE and pSPYCE; PbRD21-pSPYCE and pSPYNE) (Fig. 7b). Furthermore, PbMC1a/1b interacted with PbRD21 in vitro according to a GST pull-down assay (Fig. 7c). Y2H, BiFC and GST pull-down assays indicated that PbMC1a/1b interacted with PbRD21 and that their interaction occurred in the cytoplasm and nucleus.

### Transient expression of *PbMC1a/1b* and *PbRD21* changed the lignin content in pear fruits and fruit calli

**Fig. 8:**
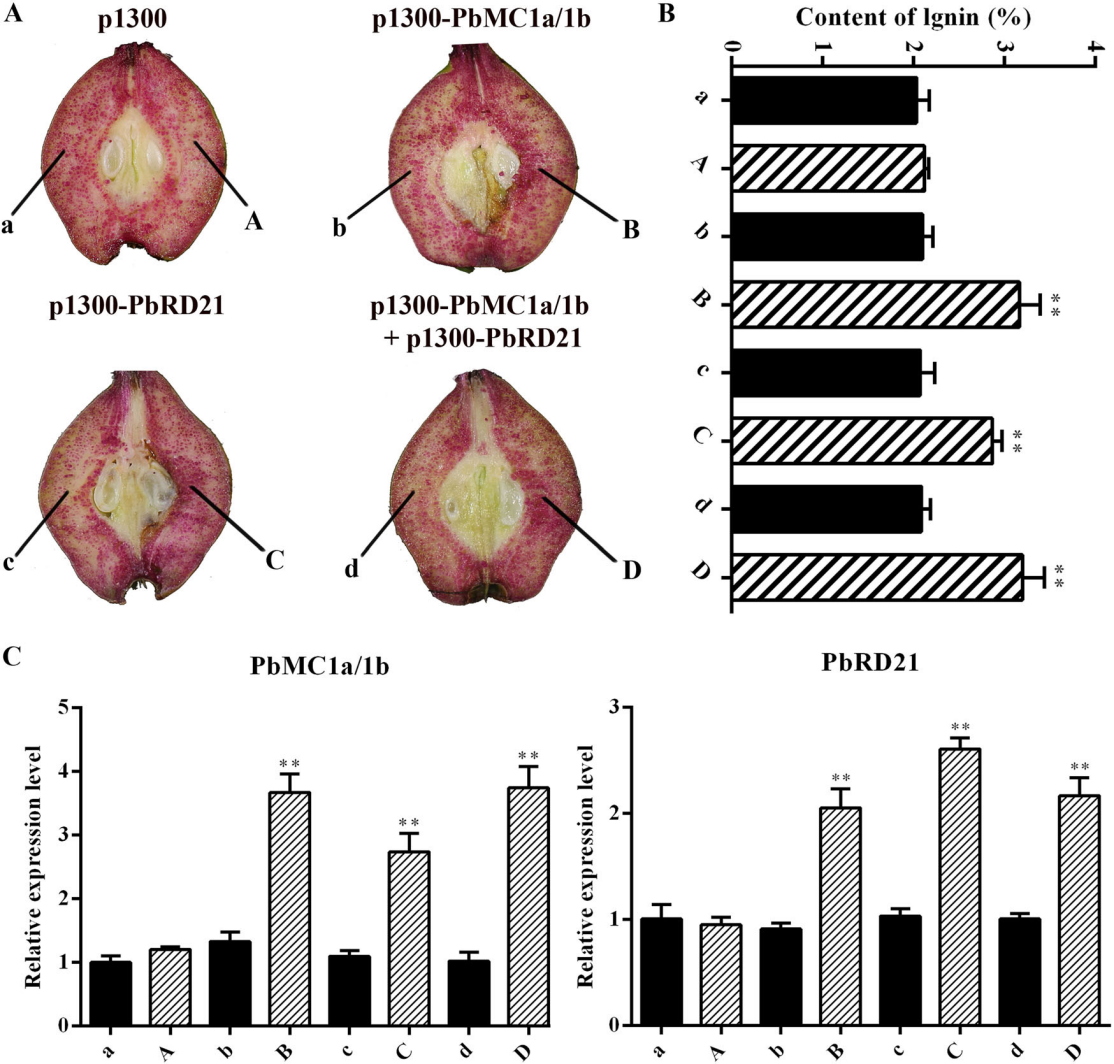
Lignin contents and gene expression levels in fruit tissues infiltrated with *PbMC1a/1b* and *PbRD21* overexpression constructs. **a** Transient assay for the overexpression of *PbMC1a/1b* and *PbRD21* in ‘Dangshan Su’ at 37 DAF. For different gene constructs, the injection sites were labeled A, B, C, and D, and the corresponding noninjected points were labeled a, b, c, and d. Images were obtained 7 days after the infiltration of *Agrobacterium*. **b** Lignin contents in the fleshy tissues in the fruits around the injection points (A, B, C, and D) and corresponding noninjected points (a, b, c, and d) (**p < 0.01). **c** The expression levels of *PbMC1a/1b* and *PbRD21* in the fleshy tissues around the injection points and corresponding noninjected points were analyzed using qRT-PCR

To verify the roles of *PbMC1a/1b* and *PbRD21* in lignin biosynthesis, overexpression vectors were transiently expressed in pear fruit. 7 days after injection, increases in lignin staining were observed at the injection sites used under the overexpression of *PbMC1a/1b*, *PbRD21* and a mixture of the two (Fig. 8a). The lignin contents were increased by 50.6, 38.2, and 52.9% compared with corresponding noninjected points, but no increase was evident in plants injected with the control vector (Fig. 8b). In addition, the injection of the *PbMC1a/1b* overexpression construct increased the expression of *PbRD21* and vice versa (Fig. 8c). At the same time, lignin contents increased significantly after the overexpression of *PbMC1a/1b* and *PbRD21* and decreased after their silencing in fruit calli (Fig. S5). Furthermore, the expression level of *PbRD21* was similar to the contents of stone cells, lignin, and *PbMC1a/1b* (Fig. S6).

## Discussion

Stone cells are widely present in various parts of fruit, especially in the flesh, which reduces the quality of fruit^34–36^. Recent studies have focused on the cloning and regulation of lignin synthesis structural genes based on the pear genome and germplasm resources^37,38^. Cell wall lignification is associated with PCD, and MCs play a key role in this process^27^. Only a few *MCs* have been characterized, mainly from model plants^21^. The functional roles and potential regulatory mechanisms of *MCs* in perennial fruit trees remain elusive. Therefore, clarifying the function of *MCs* in pear will promote our understanding of the multiple roles of *MCs*. Here, we demonstrated that *PbMC1a/1b* and its interacting protein (*PbRD21*) promoted the expression of lignin biosynthetic genes, cell lignification and, thus, likely the formation of stone cells in pear fruit.

*MCs* play a key role in plant growth, development, and biotic and abiotic stress responses^39^ and show specific expressions in different tissues and organs in *Arabidopsis*^40^, rice^41^, grape^42^, and tomato^43^. In this study, we performed genome-wide analyses and identified 11 *PbMCs*. *PbMC1a/1b*, *PbMC2b*, *PbMC3c*, *PbMC4b* and *PbMC4c* were expressed in pear fruit and may be related to fruit development. During the development of fruit, the peak lignin content appeared before the peak of stone cells^34^. The development of stone cells is a process associated with the SCW thickening and lignification of parenchyma cells, and the synthesis of lignin provides material for the development of stone cells^44^. Our results demonstrated that lignin was an important raw material for stone cell formation.

During the formation of stone cells in pear fruits, PCD and SCW thickening are accompanied by cellulose and lignin deposition^45^. Cell death is regulated by transcription during xylem maturation, including the development of the SCW and lignification of cells^46^. The sequencing of signaling mutants indicated that the expression of *MCs* interfered with the biosynthesis of lignin^47^. RNAi knockdown of *PttMC13* and *PttMC14* driven by the xylem-specific *proPtCOMT* promoter inhibits expression more effectively than that driven by the 35S promoter^27^, and *COMT* reduces the syringyl lignin content significantly when the expression of *COMT* is decreased^48^. In this study, transgenic *PbMC1a/1b Arabidopsis* grew slowly; the cell walls of the vessels, xylary fibers, and interfascicular fibers were thicker than those of the WT; and the lignin contents of the stems were increased significantly. These results demonstrated that *PbMC1a/1b* promoted the thickening of the cell walls via lignin deposition.

Most enzymes involved in lignin monomer biosynthesis, such as *C4H*, *CAD*, *CSE*, and *LAC*, have been reported^49^. The expression levels of these genes increased significantly in transgenic *PbMC1a/1b* plants, and *LACs* exhibited the highest expression levels. In *Arabidopsis*, there was no significant change in lignin content in the single *LAC11* mutant, and only a slight decrease was observed in the *LAC4 LAC11* and *LAC4 LAC17* double mutants. However, the lignin content of the *LAC4 LAC11 LAC17* triple mutant was reduced significantly, indicating that there is functional redundancy among these genes^31,33^. Furthermore, *LAC11* is the direct target of *SND1* and *VND7*, and the promoter of *LAC11* exhibits four secondary wall *NAC*-binding elements (SNBE)^50^. Interestingly, the post-translationally activated target genes of *VND6* and *VND7*, such as *XCP1*, *XCP2*, and *MCs*, are related to the formation of secondary wall but also to cell death^51,52^. The homologous genes of *VND7* or other transcription factors regulating *MCs* and *LACs* need further verification in pear. Whether *PbMCs* and *PbLACs* synergistically promote the formation of lignin in pear fruit also needs further verification.

*Arabidopsis RD21* is a pro-death cysteine protease that has an inactive form^53^. MCs may be responsible for RD21 processing after the rupture of the vacuole membrane in xylem elements. In addition, the activation of MCs has been reported to be responsible for processing RD21 or other proteases during xylem maturation^27^, but no additional studies have supported this claim. In this study, the interaction between PbMC1a/1b and PbRD21 was verified, and their interaction sites were in both the cytoplasm and nucleus, but their subcellular localizations were different. One explanation for the strong nucleo-cytoplasmic signals of the PbMC1a/1b-PbRD21 interaction could be the differential subcellular localization of PbRD21. Because pre-domain removal occurred during protein extraction from leaves, a change in the subcellular localization of RD21 occurred^54^. A more likely explanation is that PbMC1a/1b changes the subcellular localization of PbRD21. RD21 shows a change in its subcellular localization when interacting with proteins. Hs4E02 is located in the nucleus, causing RD21A to accumulate in different cell compartments, and interaction is observed in the cytoplasm as well as the nucleus^55^. AtSerpin1 is a protease that inhibits AtMC1-mediated cell death and colocalizes in the cytoplasm and nucleus^56^. RD21 is simultaneously targeted by an inhibitor of AtSerpin1. A PCD inducer increases vesicle membrane permeability and leads to the co-localization of RD21 and AtSerpin1 in the cytoplasm^57,58^. Collectively, our data demonstrated that PbMC1a/1b interacts with PbRD21.

Lignin contents in pear fruits and flesh calli were also changed significantly by the transient expression of *PbMC1a/1b* and *PbRD21*, and their expression levels were positively correlated with the lignin content. However, the up- or downregulated contents of lignin biosynthesis genes were similar in *PbMC1a/1b* and *PbRD21* single or co-transformed materials. Although caspase and MCs have different substrate specificities and biochemical properties, they are functionally similar: both proteases can hydrolyze a single conserved substrate^53^. They may compete for substrates and result in similar upregulation of lignin contents in single or multiple mutants. MCs may be involved in postmortem autolysis, for example, through interacting with RD21 and other proteolytic enzymes. How MCs interact with RD21 or other proteolytic enzymes to promote lignin deposition has not been reported until now; however, the mechanism of their interaction needs further study.

## Conclusion

Overall, our results demonstrated that *PbMC1a/1b* (PCD-associated gene) promoted SCW thickening and increased the expression levels of lignin biosynthetic genes and lignin contents and clarified the involvement of *PbMC1a/1b* in pear fruit cell lignification. The overexpression of *PbMC1a/1b* led to high expression of *LACs*, but further research is needed to identify the synergy between them. The cysteine protease RD21, a putative target protein of MCs, is another protease associated with PCD and was identified as a member of the interaction. *PbMC1a/1b* and *PbRD21* could promote lignin formation, and the mechanism of their interaction requires further exploration. These studies will help reveal the mechanism of SCW thickening and lignin deposition to form stone cells in pear fruits from the perspective of PCD. In the future, a single-cell approach may be needed to decipher the regulatory mechanisms of *MCs*, *RD21*, and PCD at the early stage of lignification, to identify the different activities between parenchyma cells and lignifying cells and to prevent the formation of stone cells in pear fruit.

## Materials and methods

### Plant materials

The study was conducted in a 50-year-old ‘Dangshan Su’ Pear (*P. bretschneideri*) orchard in Xiaji Town, Gaoyou City, Jiangsu Province, and healthy fruit trees were selected. One hundred fruit samples were collected on ice at 15, 22, 30, 37, 45, 60, and 75 DAF. Fruits were chopped after peeling, some flesh was used for the determination of physiological indicators, and the remaining flesh samples were frozen in liquid nitrogen and stored in a refrigerator at −80 °C for subsequent tests. Fresh root, stem, leaf, pollen and pulp tissues, and organs were sampled from the same plant, quickly frozen in liquid nitrogen and stored at −80 °C. The wild-type of *A. thaliana* was the Columbia ecotype.

### Stone cell content in pear flesh

Stone cell content analysis was performed based on the method of Syros et al.^59^. The operation steps were as follows: twenty fruits at 15, 22, 30, 37, and 45 DAF and three fruits at 60 and 75 DAF of uniform sizes were selected and peeled, and the edible parts of the fruits were reserved for testing. One hundred grams of flesh was frozen at −20 °C for 24 h, then thawed at room temperature, and homogenized with distilled water in a blender for 10 min, after which the sample was stirred for 1 min, allowed to stand for 3 min, and the upper layer suspension was discarded. The remainder of the suspension was precipitated with 0.5 mM hydrochloric acid (HCl) for 30 min, decanted and washed with distilled water. The procedures were repeated several times until there were only stone cells.

### Lignin determination

Twenty fruits at 15, 22, 30, 37, and 45 DAF, three fruits at 60 and 75 DAF, twenty *Arabidopsis* stems and twenty fruit calli were oven dried. After being ground to powder, 0.01 g of the samples with 3 replicates per sample was used to determine lignin content according to the method described by Tao et al.^5^. Samples were ground with 95% ethanol, washed 3 times with 95% ethanol and ethanol:hexane (1:2, v/v), and dried. The dried precipitates were digested in 2 ml of 25% (v/v) acetyl bromoacetic acid solution and crystallized at 70 °C for 30 min. The reaction was terminated by the addition of 0.9 ml of 2 N NaOH, after which 5 ml of acetic acid and 0.1 ml of 7.5 M hydroxylamine hydrochloride were added. The volume was adjusted to 10 ml with acetic acid, and the absorbance at A280 was determined. Lignin contents were calculated from a lignin standard sample (Sigma-Aldrich, USA) curve.

### Identification of *PbMCs* genes in the white pear genome and bioinformatics analyses

The general feature formats (GFFs), proteome sequences and complete genome of pear (*P. bretschneideri*) were downloaded from http://peargenome.njau.edu.cn^28^. The protein sequences of *Arabidopsis* were obtained from http://www.arabidopsis.org/^60^. If two or more protein sequences at the same locus were identical in two proteome datasets, the longest sequence was selected where they overlapped. The HMM configuration file for the NAM domain (PF00656) was downloaded from http://pfam.xfam.org/family/PF00656/. HMMER was used to search a custom database containing proteomes with a threshold set to the Pfam GA aggregation cutoff value^61^. Proteins selected by HMMER were used for BLASTp queries in the original protein database. The NAM domains of the BLASTp hits were scanned using InterPro (http://www.ebi.ac.uk/interpro/)^62^, and those showing an E-value less than 1e−10 and the protein domains were considered. *PbMCs* genes were named based on homologous genes in *Arabidopsis*. Finally, a phylogenetic tree was constructed by the maximum likelihood (ML) method using MEG 6.0.

### RNA extraction, semiquantitative RT-PCR and qRT-PCR analyses

Total RNA was extracted with the Polysaccharide Polyphenol Plant RNA Extraction Kit (Chengdu Fuji Biotechnology, China). According to the instruction manual, ~1 µg of total RNA was transcribed into cDNA using HiScript Q RT SuperMix for qPCR (+gDNA wiper) (Vazyme, China). The primer sequences for the semiquantitative RT-PCR and qRT-PCR assays were designed using Primer 5 (in the program EMBOSS Explorer) and were listed in Table S3. The method and program of Shi et al.^63^ were employed for semiquantitative RT-PCR. qRT-PCR was performed using a SYBR® Green Premix kit (TaKaRa Biotechnology, China). The program of Jin et al.^64^ was used for qRT-PCR. Each cDNA was analyzed in triplicate, after which the average threshold cycle (Ct) was calculated for each sample. Relative expression levels were calculated with the 2^−ΔΔCt^ method^65^. *Tubulin* was analyzed in parallel as a reference control for *P. bretschneideri*, and *AtActin* was used for *Arabidopsis*.

### Gene cloning and vector construction

The full-length *PbMC1a/1b* without the stop codon was cloned by RT-PCR with primer pairs containing *Xba* I and *BamH* I restriction sites (Table S3) using the Phanta® Max High-Fidelity PCR Enzyme (Vazyme, China). The product was introduced to the pCAMBIA1300 vector to generate a fusion construct (p35S-*PbMC1a/1b*-GFP) using a One-step Rapid Cloning Kit (Vazyme, China). The same methods were used for cloning and introduction into the pTRV2 vector (containing *Xba* I and *Sac* I restriction sites) to generate the pTRV2-*PbMC1a/1b* fusion construct. The fusion constructs (p35S-*PbMC1a/1b*-GFP and pTRV2-*PbMC1a/1b*) were transferred to *E. coli* strain DH5α by a freeze-thaw method. After sequence confirmation, the fusion constructs and control vector (pCAMBIA1300) were transferred to *Agrobacterium tumefaciens* strain GV3101 via the same freeze-thaw method. Finally, the same methods were used to construct the p35S-*PbRD21*-GFP and pTRV2-*PbRD21* fusion expression vectors.

### Subcellular localization of *PbMC1a/1b* and *PbRD21*

Transient transformation of tobacco leaves was performed using the method described by Yang et al.^66^. *Agrobacterium* was cultured at a ratio of 1:100 in LB liquid medium (containing 50 μg/mL kanamycin and 50 μg/mL rifampicin)^64^. The bacterial suspension was incubated at 28 °C on a 200 r/min shaker and grown to an OD600 of 0.6–0.8. The cells were collected by centrifugation at 4500 × *g* for 10 min and resuspended in a solution containing 10 mmol/L MgCl_2_, 10 mmol/L MES and 0.15 mmol/L AS. The bacterial suspension liquid was adjusted to an OD600 of 0.8–1.2 and cultured for 3 h in the dark. The inoculum was injected into the lower surface of 5-week-old *N. benthamiana* leaves. The transformed leaves were grown for 48 h and then subjected to live cell imaging using an inverted confocal microscope (Zeiss LSM 780, Germany).

### Transient expression of pear fruit and fruit calli

*Agrobacterium* cells were collected and activated using the same method as that used for subcellular localization. Cells were injected into the flesh of ‘Dangshan Su’ pears at 37 DAF using needleless syringes. Six fruits were injected with each construct. The transformed fruits were placed in a dark environment at 25 °C overnight and then transferred to a growth chamber (25 °C, 16 h light/8 h dark photoperiod) for 7 days under low light conditions. Thereafter, the fruits were stained with phloroglucinol-HCl (Wiesner reagent)^29^, and lignin contents were determined. The expression levels of genes were determined by qRT-PCR, and the primers were listed in Table S3. Twenty fruit calli were also soaked in the *Agrobacterium* suspensions, cultured for 1 h in the dark, and placed on MS medium without growth regulators for 7 d in the dark, after which they were dried, and the lignin content was determined as previously described.

### Transformation and characterization of transgenic plants

*Arabidopsis* plants (ecotype Columbia) were transformed by the vacuum infiltration of whole plants in an *Agrobacterium* suspension according to Bechtold et al.^67^. The plants were self-pollinated, and the seeds were harvested after the stems were completely dry. The T1 generations were selected on *Arabidopsis* medium containing 20 mg/L hygromycin^36^. After transgenic *Arabidopsis* seedlings were obtained, genomic DNA was extracted from in vitro-grown T1 and WT plants using cetyltrimethyl ammonium bromide (CTAB)^36^. To verify the transgenic *Arabidopsis* plants, PCR assays were performed, in which each 20 μl reaction contained 10 μl of buffer (Vazyme, China), 1 μl of DNA, 1 μl of each primer and 7 μl of sterile distilled water. The conditions described for PCR and electrophoresis in agarose gels by Jin et al.^63^ were followed. The relative expression levels of *PbMC1a/1b* in the leaves of T1 plants were determined by qRT-PCR with three technical replicates for each sample. The line showing the highest expression among the three lines was selected for subsequent verification, and the T3 generation of homozygous *Arabidopsis* was used for functional verification.

Transgenic T3 generation and WT plants were transferred to MS medium without growth regulators. The root lengths of 50 seedlings from each line were measured after 1 week of germination. Seedlings were transplanted into plastic pots with a mixture of vermiculite and soil (1:2) under 16 h of light and 40% relative humidity in the same chamber. The plant heights of 30 plants for each line were determined at 5 and 8 weeks. Stems were harvested at 8 weeks^68^, and toluidine blue staining, which was repeated three times for each line, was performed using the method of Berthet et al.^31^. ImageJ software was used to measure stem diameters as well as the cell wall thicknesses of vessels, xylary fibers, and interfascicular fibers in 100 cells. Lignin contents in the stems of 20 plants were determined at the mature stage. RNAs were extracted from the stems of the WT and transgenic T3 generations, and the expression levels of genes involved in phenylpropanoid biosynthesis (pxb00940) were quantitatively determined by qRT-PCR (primers listed in Table S3).

### Y2H and BiFC assays

To explore the molecular mechanism whereby the overexpression of *PbMC1a/1b* to increases lignin content, the full-length cDNA of PbRD21 was amplified using a primer pair (Table S3) and cloned into the pGADT7 vector to obtain AD-PbRD21. PbMC1a/1b was amplified with primers (Table S3) and inserted into pGBDT7 to generate BD-PbMC1a/1b. The two constructs were transformed together into AH109 yeast cells, followed by culturing in SD/-Ade/-His/-Leu/-Trp plates to identify DNA-protein interactions.

For BiFC analysis, *PbRD21* without a stop codon was cloned with a primer pair (Table S3) and introduced into pUC-pSPYCE-35S(nYFP) to obtain PbRD21-nYFP. Simultaneously, *PbMC1a/1b* without a stop codon was amplified using a primer pair (Table S3) and inserted into pUC-pSPYNE-35S(cYFP) to produce PbMC1a/1b-cYFP. Transient expression analyses of PbMC1a/1b-cYFP and PbRD21-nYFP in tobacco leaves were carried out according to Zhao et al.^33^. YFP fluorescence was observed using a confocal laser scanning microscope (LSM510 Meta, Zeiss, Germany).

### In vitro GST pull-down assay

The GST-PbMC1a/1b and His-PbRD21 constructs were introduced into the *E. coli* BL21 (DE3) strain. One mM isopropyl sulfo-beta-galactoside (IPTG) was added at 37 °C to induce the expression of the fusion proteins. Glutathione-agarose 4B (GE Healthcare) beads and Ni-agarose were used according to the manufacturer’s instructions (GE Healthcare) to purify GST-PbMC1a/1b and His-PbRD21. For the protein pull-down assay, the GST fusion proteins were combined in a glutathione Sepharose 4B column. The loaded matrix was incubated with the purified His fusion protein in binding buffer for 4 h at 4 °C. The beads were centrifuged at 2000 × *g* for 1 min at 4 °C and washed three times with protein pull-down wash buffer, and the bound proteins were eluted and fractionated by 10% SDS-PAGE.

### Statistical analysis

Each treatment was repeated at least three times with consistent results. Data are presented as the means ± SE of at least three independent replicates from one representative experiment. The data were analyzed with the ANOVA program of SPSS (IBM SPSS 22), where **P < 0.01 was considered to indicate a significant difference.

## Supplementary information

Supplementary TableS1

Supplementary TableS2

Supplementary TableS3

Supplementary FigureS1

Supplementary FigureS2

Supplementary FigureS3

Supplementary FigureS4

Supplementary FigureS5

Supplementary FigureS6
