# Supplementary material for: PbMC1a/1b regulates lignification during stone cell development in pear (Pyrus bretschneideri) fruit
Source: Hortic Res. 2020 May 1;7:59. doi: 10.1038/s41438-020-0280-x (PMC7193627; doi:10.1038/s41438-020-0280-x)
Supplement: Supplementary file 1 — Supplementary TableS1 [file 41438_2020_280_MOESM1_ESM.docx]

**Table S1.** Results of BLAST search using the AtMC protein sequences in the Chinese white pear genome database.

|  |  |  | full sequence | | | best 1 domain | | | domain number estimation | | | | | | | |  |
| --- | --- | --- | --- | --- | --- | --- | --- | --- | --- | --- | --- | --- | --- | --- | --- | --- | --- |
| target name | query name | accession | E-value | score | bias | E-value | score | bias | exp | reg | clu | ov | env | dom | rep | inc | description of target |
| Pbr001381.1 | Peptidase_C14 | PF00656.18 | 1.40E-111 | 373.7 | 0.2 | 2.40E-59 | 202.5 | 0.0 | 2.0 | 2 | 0 | 0 | 2 | 2 | 2 | 2 | Protein |
| Pbr002543.1 | Peptidase_C14 | PF00656.18 | 6.80E-80 | 269.8 | 0.0 | 9.50E-80 | 269.4 | 0.0 | 1.2 | 1 | 0 | 0 | 1 | 1 | 1 | 1 | Protein |
| Pbr010269.1 | Peptidase_C14 | PF00656.18 | 2.20E-79 | 268.2 | 0.0 | 3.00E-79 | 267.7 | 0.0 | 1.2 | 1 | 0 | 0 | 1 | 1 | 1 | 1 | Protein |
| Pbr010259.1 | Peptidase_C14 | PF00656.18 | 5.60E-79 | 266.8 | 0.0 | 7.20E-79 | 266.5 | 0.0 | 1.1 | 1 | 0 | 0 | 1 | 1 | 1 | 1 | Protein |
| Pbr002869.1 | Peptidase_C14 | PF00656.18 | 1.80E-61 | 209.5 | 0.0 | 2.30E-61 | 209.1 | 0.0 | 1.1 | 1 | 0 | 0 | 1 | 1 | 1 | 1 | Protein |
| Pbr018439.1 | Peptidase_C14 | PF00656.18 | 2.10E-58 | 199.5 | 0.0 | 3.80E-58 | 198.6 | 0.0 | 1.5 | 1 | 1 | 0 | 1 | 1 | 1 | 1 | Protein |
| Pbr028452.1 | Peptidase_C14 | PF00656.18 | 1.30E-51 | 177.2 | 0.1 | 2.20E-51 | 176.5 | 0.0 | 1.3 | 2 | 0 | 0 | 2 | 2 | 2 | 1 | Protein |
| Pbr003790.1 | Peptidase_C14 | PF00656.18 | 1.30E-50 | 174 | 0.1 | 2.10E-50 | 173.2 | 0.0 | 1.3 | 2 | 0 | 0 | 2 | 2 | 2 | 1 | Protein |
| Pbr042892.1 | Peptidase_C14 | PF00656.18 | 8.50E-39 | 135.2 | 0.0 | 1.50E-38 | 134.5 | 0.0 | 1.3 | 1 | 0 | 0 | 1 | 1 | 1 | 1 | Protein |
| Pbr012828.1 | Peptidase_C14 | PF00656.18 | 2.80E-38 | 133.5 | 0.0 | 4.50E-38 | 132.8 | 0.0 | 1.4 | 1 | 1 | 0 | 1 | 1 | 1 | 1 | Protein |
| Pbr019077.1 | Peptidase_C14 | PF00656.18 | 2.80E-38 | 133.5 | 0.0 | 4.50E-38 | 132.8 | 0.0 | 1.4 | 1 | 1 | 0 | 1 | 1 | 1 | 1 | Protein |
| Pbr031812.1 | Peptidase_C14 | PF00656.18 | 5.50E-33 | 116.2 | 0.0 | 7.80E-33 | 115.7 | 0.0 | 1.2 | 1 | 0 | 0 | 1 | 1 | 1 | 1 | Protein |
| Pbr029279.1 | Peptidase_C14 | PF00656.18 | 0.0045 | 18.2 | 0.0 | 0.011 | 17 | 0.0 | 1.5 | 1 | 1 | 0 | 1 | 1 | 1 | 1 | Protein |
| Pbr023157.1 | Peptidase_C14 | PF00656.18 | 0.0082 | 17.4 | 0.0 | 0.012 | 16.8 | 0.0 | 1.4 | 1 | 1 | 0 | 1 | 1 | 1 | 1 | Protein |
| Pbr019817.1 | Peptidase_C14 | PF00656.18 | 0.0085 | 17.3 | 0.0 | 0.0099 | 17.1 | 0.0 | 1.4 | 1 | 1 | 0 | 1 | 1 | 1 | 1 | Protein |
