# Supplementary material for: PbMC1a/1b regulates lignification during stone cell development in pear (Pyrus bretschneideri) fruit
Source: Hortic Res. 2020 May 1;7:59. doi: 10.1038/s41438-020-0280-x (PMC7193627; doi:10.1038/s41438-020-0280-x)
Supplement: Supplementary file 2 — Supplementary TableS2 [file 41438_2020_280_MOESM2_ESM.docx]

**Table S2.** Basic information of *MC* genes in *Pyrus bretschneideri.*

| Gene name | Gene ID | Chr(Mbp) | Start | End | CDS length(bp) | Genomic sequence length(bp) | Protein length(aa) | Molecular weight(Da) | PI |
| --- | --- | --- | --- | --- | --- | --- | --- | --- | --- |
|  |  |  |  |  |  |  |  |  |  |
|  |  |  |  |  |  |  |  |  |  |
| *PbMC1a* | *Pbr012828.1* | scaffold197.0(0.7) | 64955 | 66510 | 717 | 1556 | 239 | 24941.8 | 4.81 |
| *PbMC1b* | *Pbr019077.1* | Chr9(22.4) | 1227816 | 1229371 | 717 | 1556 | 239 | 24941.8 | 4.81 |
| *PbMC1c* | *Pbr018439.1* | Chr1(10.7) | 2902488 | 2905874 | 1173 | 3387 | 391 | 41213.7 | 6.34 |
| *PbMC2a* | *Pbr001381.1* | Chr12(22.8) | 18639715 | 18643850 | 1845 | 4136 | 615 | 67589.7 | 7.53 |
| *PbMC2b* | *Pbr002869.1* | Chr7(15.3) | 12105220 | 12108178 | 1089 | 2959 | 363 | 39729 | 6.63 |
| *PbMC3a* | *Pbr028452.1* | scaffold471.0.1(0.4) | 176276 | 178082 | 1074 | 1807 | 358 | 39395.8 | 8.55 |
| *PbMC3b* | *Pbr003790.1* | scaffold1174.0(0.1) | 48011 | 49757 | 1074 | 1747 | 358 | 39568 | 8.67 |
| *PbMC3c* | *Pbr042892.1* | Chr5(28.4) | 1597383 | 1608764 | 996 | 11382 | 332 | 37098.7 | 8.21 |
| *PbMC4a* | *Pbr002543.1* | Chr5(28.4) | 22013999 | 22016502 | 1353 | 2504 | 451 | 49188.7 | 4.91 |
| *PbMC4b* | *Pbr010259.1* | Chr10(26.2) | 1049256 | 1051786 | 1248 | 2531 | 416 | 45266.7 | 5.62 |
| *PbMC4c* | *Pbr010269.1* | Chr10(26.2) | 1149741 | 1151938 | 1248 | 2198 | 416 | 45417.8 | 5.41 |
