# Supplementary material for: PbMC1a/1b regulates lignification during stone cell development in pear (Pyrus bretschneideri) fruit
Source: Hortic Res. 2020 May 1;7:59. doi: 10.1038/s41438-020-0280-x (PMC7193627; doi:10.1038/s41438-020-0280-x)
Supplement: Supplementary file 3 — Supplementary TableS3 [file 41438_2020_280_MOESM3_ESM.docx]

**Table S3.** Sequences for the gene-specific primers used in this work.

| **Gene ID** | **Forward primer** | **Reverse primer** |
| --- | --- | --- |
| PbMC1a | GGCGATGAAGTTGATGGATATG | TATCGTCCACTCCTGTCCATTC |
| PbMC1b | GGCGATGAAGTTGATGGATATG | TATCGTCCACTCCTGTCCATTC |
| PbMC1c | TATGCTGGTGAATTGCTGCCACT | GCGTGAGAGTAAGGAGCTGGA |
| PbMC2a | CTGGTGCCATGACTTATGCTTTCA | CGAGTACGGGGCTGTATAGGTG |
| PbMC2b | GCTCACCGAAGAAGAAGTTGATCC | TGCCCCAGCAGGTAGAGGTC |
| PbMC3a | CGGCTTTATCTGGAGGCACTTC | CGTTGCTGCCTAATACTGGCAG |
| PbMC3b | ATGGCATGTCGGAATGAGAGG | GGGCCTGGACATCACTCTAGT |
| PbMC3c | AAGCAATGGGTTCAAATGACGC | CCTTGGTTTGAAAACACCTGCC |
| PbMC4a | GGCAGTGGCAGTTTTGGTGGA | TGCCCAACATCAATGTCGTCCT |
| PbMC4b | CATCACAATGCAATTCTCCTTCTC | TTTTTCCGGTGGGCTGAGTG |
| PbMC4c | GGCGGAAGATGCACTGGGAT | TGTCATCCTTGCCTGTTTTCTGC |
| Actin | TACTCTTTCACCACAACTGC | CTCGTAACTCTTCTCCACAG |
| TUB | TGGGCTTTGCTCCTCTTAC | CCTTCGTGCTCATCTTACC |
| pCM1300-PbMC1a/b | gagaacacgggggactctagaATGGCATTATATTGGCTTGTACAAG | gcccttgctcaccatggatccTAGGGAGAAGGGTTTTGCATACA |
| pCM1300-PbRD21 | gagaacacgggggactctagaATGTCCATCGTCGACTACAACG | gcccttgctcaccatggatccAGCACTTGGGAAGCGGAAAC |
| AtAct | AGGCACCTCTTAACCCTAAAGC | GGACAACGGAATCTCTCAGC |
| PAL1 | AGTTTGGATTATGGATTCAAGGGA | TCAGAAGTTTTGCGAGACGAGAT |
| C4H | ATCTCAACCACCGTAATCTCGTC | CGCCGTAAACAGTGAACACCATA |
| 4CL1 | AGAACCCGAATCTTTATTTCCACAG | AGCCACCGTCACTTTACACCTC |
| C3H1 | TTCTAATAGCGGTGGCGACAA | GAGCCCACTCGTAGTAACATCTGA |
| CCoAOMT1 | AAGACAACTACATCAACTACCACAAGC | CAGGAGGAGCCACGACAGAAC |
| COMT | CTTCCGTTCTTCCGATGGCT | GAAAGTTTACGGTTGGAGCAGG |
| HCT | GCCACCGAGACACCAATCACT | AGAACACCAGCACCGTTACAATC |
| F5H1 | CCCGTGACAATATCAAAGCAATC | CTCCGTAATAACTCCGTTAAGGC |
| CCR1 | AATCCAGATGATCCGAAGAACAC | TAAGAGCCTCGTAGTCCTGAAGA |
| CAD4 | TAAGGAGCACAGGAGCAGATGAT | CCAACACCTCACCTACTACCTCG |
| CAD9 | TTGGGATCGCTACAAAAGTTGGT | GTTCTCCGAATAGCCACCGTAA |
| LAC4 | TATCCAGGTCCCACAATCTACG | GGCACTGAGTTATGTAAGCAGGC |
| LAC11 | GCGGTCTACACTAAACCATTCACA | TTTGTTGTCTACGGATACAGGAGC |
| LAC17 | CAACTGCTCCGCAAAAGATACAT | GATGGTTTCAGTCTCAAATGGCTTA |
| BD-PbMC1a/b | CATATGGCCATGGAGGCCGAATTCATGGCATTATATTGGCTTGTACAAG | TAGTTATGCGGCCGCTGCAGGTCGACTAGGGAGAAGGGTTTTGCATACA |
| AD-PbRD21 | ATGGCCATGGAGGCCAGTGAATTCATGTCCATCGTCGACTACAACG | ATCTGCAGCTCGAGCTCGATGGATCCAGCACTTGGGAAGCGGAAAC |
| PbMC1a/b-YNE | gagaacacgggggactctagaATGGCATTATATTGGCTTGTACAAG | gacagtactatcgatggatccTAGGGAGAAGGGTTTTGCATACA |
| PbRD21-YCE | gagaacacgggggactctagaATGTCCATCGTCGACTACAACG | gacagtactatcgatggatccAGCACTTGGGAAGCGGAAAC |
| PbRD21 | ATGAGAAGTCGTTGAAGAAGGCTG | CAGTGAAGACACCCGATTGGTAG |
| PbMC1a/b-GST | GATCTGGTTCCGCGTGGATCCATGGCATTATATTGGCTTGTACAAG | CTCGAGTCGACCCGGGAATTCTAGGGAGAAGGGTTTTGCATACA |
| PbRD21-HIS | ATGGAGCTCGGTACCCTCGAGATGTCCATCGTCGACTACAACG | AGCAGAGATTACCTATCTAGAAGCACTTGGGAAGCGGAAAC |
