# Supplementary figures and images for: PbMC1a/1b regulates lignification during stone cell development in pear (Pyrus bretschneideri) fruit
Source: Hortic Res. 2020 May 1;7:59. doi: 10.1038/s41438-020-0280-x (PMC7193627; doi:10.1038/s41438-020-0280-x)

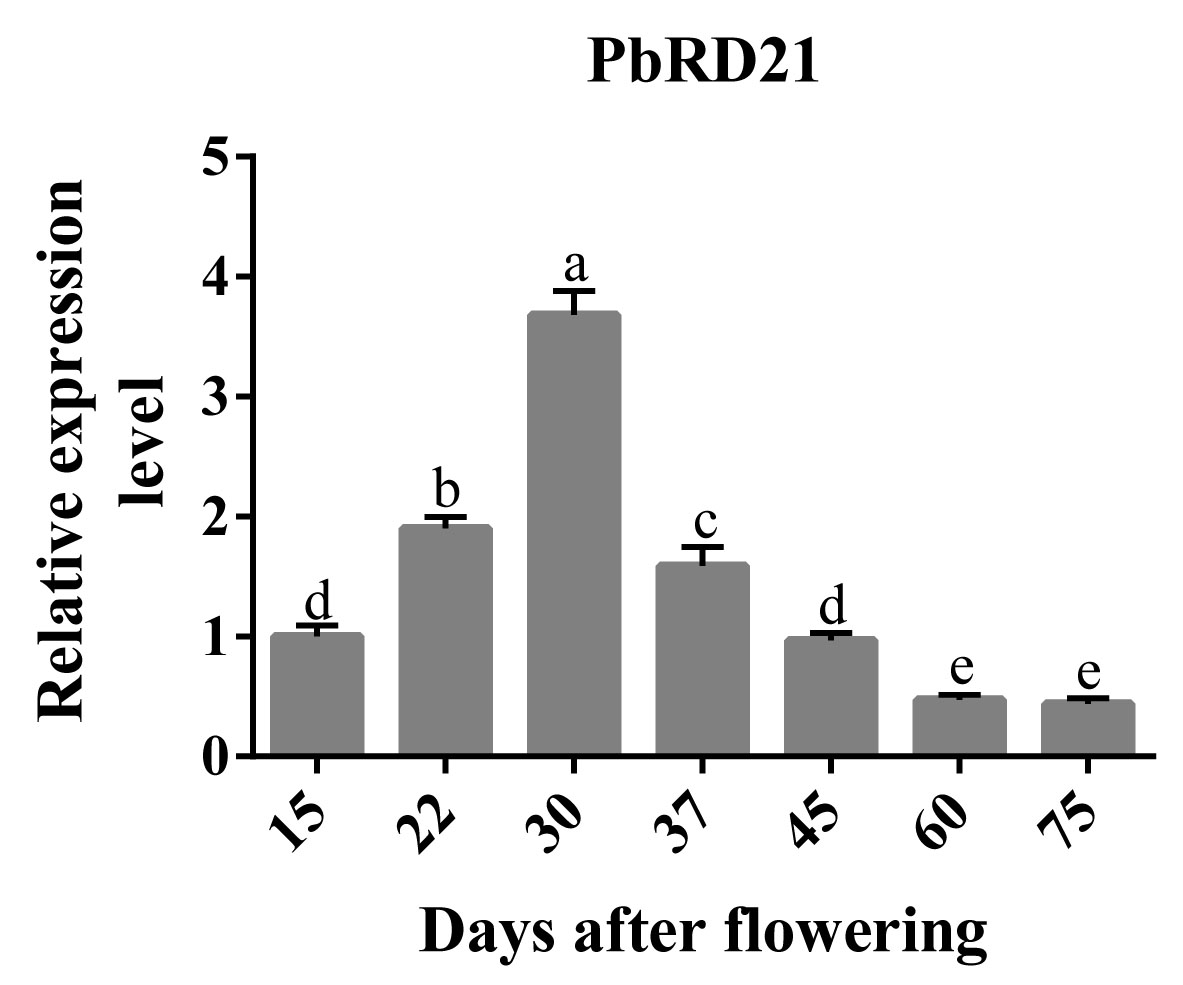

Supplement: Supplementary file 4 — Supplementary FigureS1 [file 41438_2020_280_MOESM4_ESM.jpg]

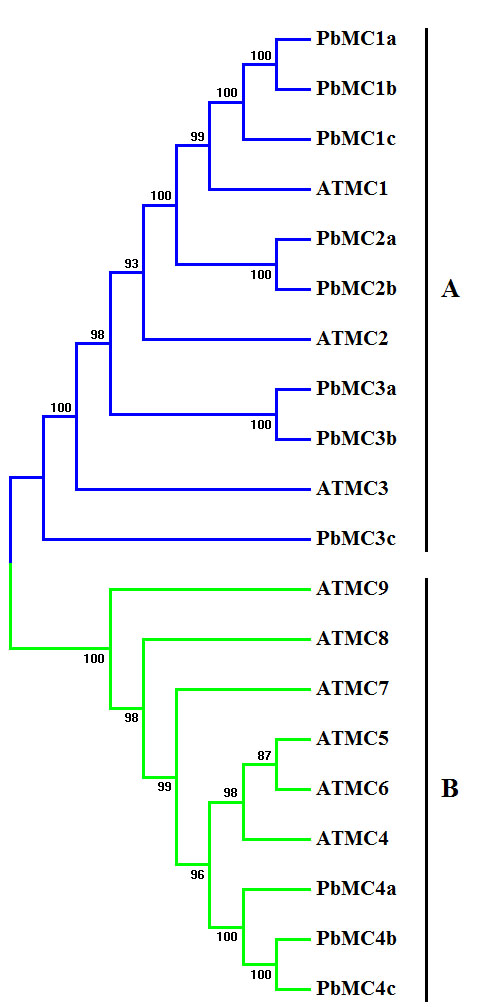

Supplement: Supplementary file 5 — Supplementary FigureS2 [file 41438_2020_280_MOESM5_ESM.jpg]

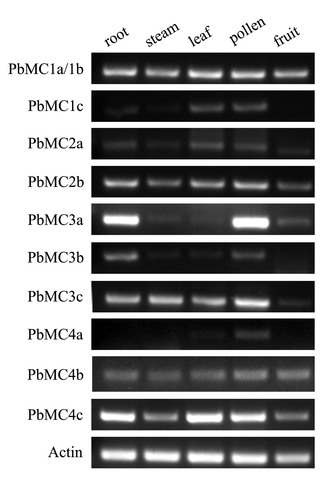

Supplement: Supplementary file 6 — Supplementary FigureS3 [file 41438_2020_280_MOESM6_ESM.jpg]

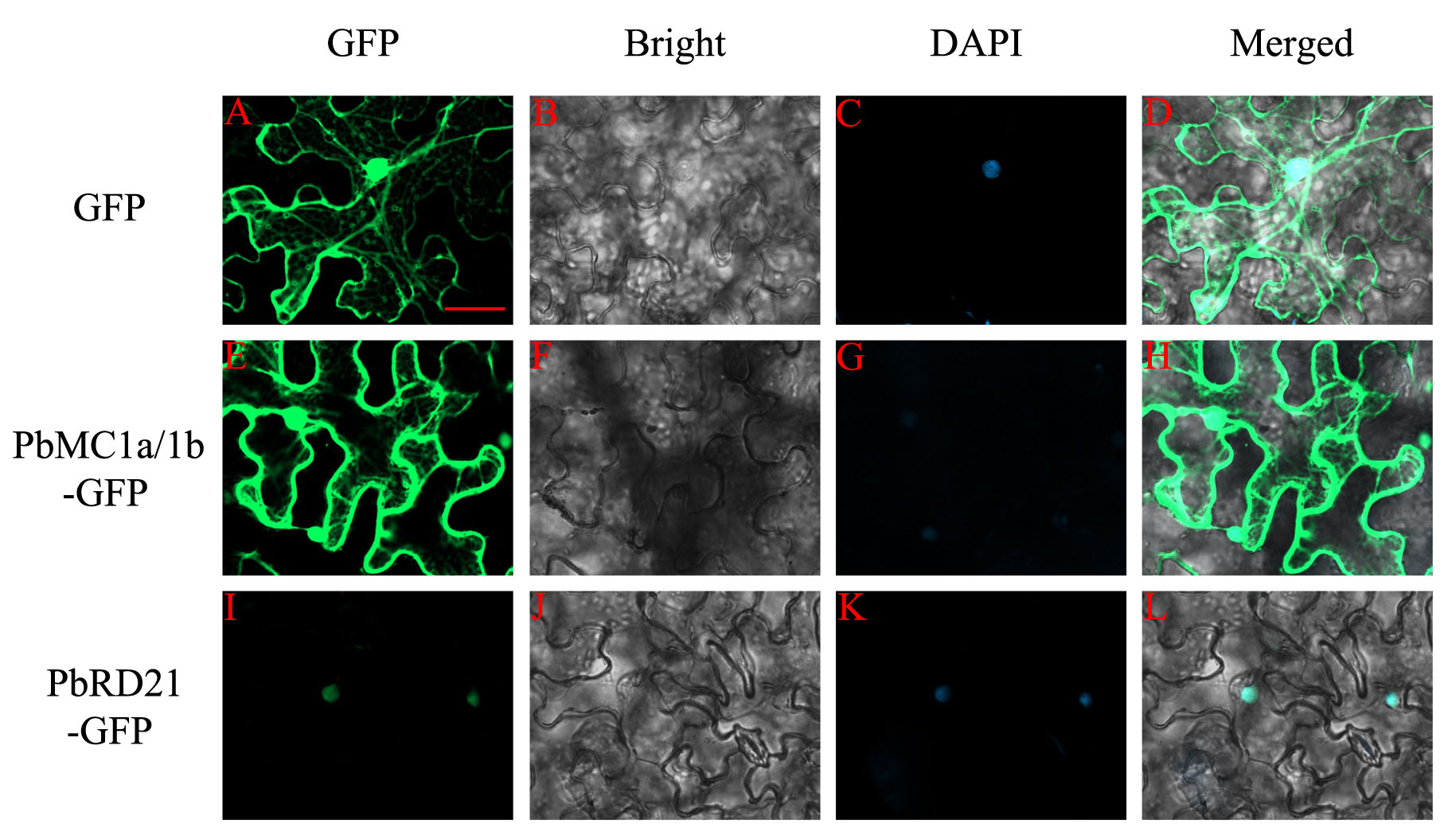

Supplement: Supplementary file 7 — Supplementary FigureS4 [file 41438_2020_280_MOESM7_ESM.jpg]

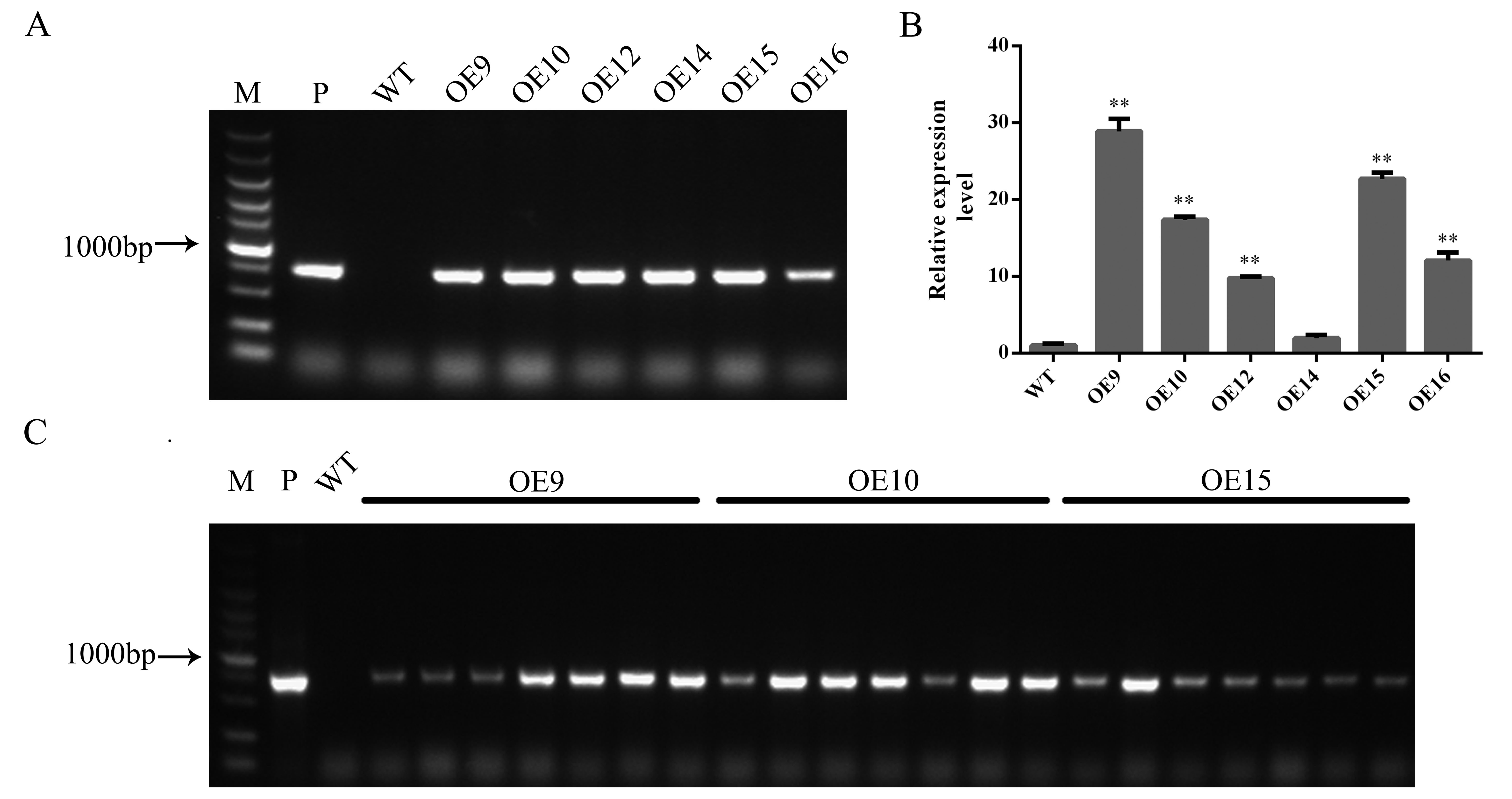

Supplement: Supplementary file 8 — Supplementary FigureS5 [file 41438_2020_280_MOESM8_ESM.jpg]

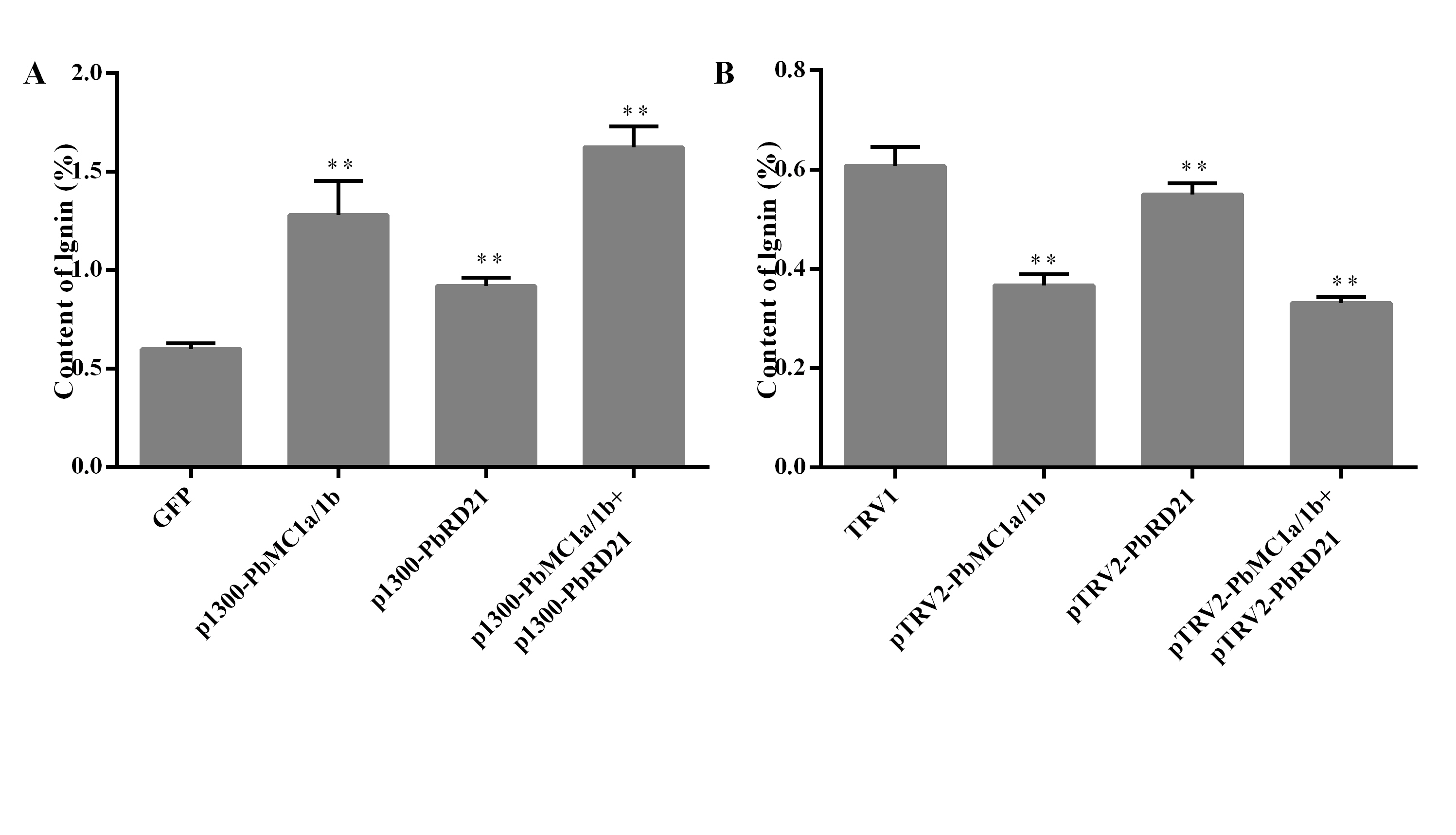

Supplement: Supplementary file 9 — Supplementary FigureS6 [file 41438_2020_280_MOESM9_ESM.jpg]
